# Regular lung recruitment maneuvers during high-frequency oscillatory ventilation in extremely preterm infants: a randomized controlled trial

**DOI:** 10.1186/s12887-022-03780-7

**Published:** 2022-12-12

**Authors:** Tobias Werther, Erik Kueng, Lukas Aichhorn, Linda Pummer, Katharina Goeral, Angelika Berger, Michael Hermon, Katrin Klebermass-Schrehof

**Affiliations:** Department of Pediatrics and Adolescent Medicine, Division of Neonatology, Pediatric Intensive Care and Neuropediatrics, Comprehensive Center for Pediatrics, Medical University of Vienna, Vienna, Austria

**Keywords:** Neonatal intensive care unit, Extremely premature infant, Oxygen Saturation, Lung volume, High-frequency ventilation, Respiratory distress syndrome, Lung recruitment

## Abstract

**Background:**

Lung recruitment maneuvers (LRMs) improve lung volume at initiation of high-frequency oscillatory ventilation (HFOV), but it is unclear when to repeat LRMs. We evaluated the efficiency of scheduled LRMs.

**Methods:**

In a randomized controlled trial, extremely preterm infants on HFOV received either LRMs at 12-hour intervals and when clinically indicated (intervention) or only when clinically indicated (control). The primary outcome was the cumulative oxygen saturation index (OSI) over HFOV time, limited to 7 days. Additionally, LRMs were analyzed with respect to OSI improvement.

**Results:**

Fifteen infants were included in each group. The mean (SD) postmenstrual age and weight at HFOV start were 23 + 6 (0 + 5) weeks and 650 (115) g in the intervention group and 24 + 4 (0 + 6) weeks (*p* = 0.03) and 615 (95) g (*p* = 0.38) in the control group. The mean (SD) cumulative OSI amounted to 4.95 (1.72) in the intervention versus 5.30 (2.08) in the control group (*p* = 0.61). The mean (SD) number of LRMs in 12 h was 1.3 (0.2) in the intervention versus 1.1 (0.5) in the control group (*p* = 0.13). Performing LRM when FiO2 > 0.6 resulted in a mean OSI reduction of 3.6.

**Conclusion:**

Regular versus clinically indicated LRMs were performed with equal frequency in preterm infants during HFOV, and consequently, no difference in lung volume was observed. LRMs seem to be most efficient at high FiO2.

**Trial registration:**

ClinicalTrials.gov ID: NCT04289324 (28/02/2020).

**Supplementary Information:**

The online version contains supplementary material available at 10.1186/s12887-022-03780-7.

## Background

Lung protective strategies are claimed to be key elements in the treatment of extremely preterm infants [1]. The first-line strategy comprises non-invasive respiratory support, in particular, continuous positive airway pressure and early surfactant administration [2]. Nevertheless, respiratory failure occurs in more than 50% of extremely preterm infants, subsequently requiring mechanical ventilation [3, 4]. Positive pressure ventilation exposes the immature lungs to lung injuries representing a major cause of morbidity in this vulnerable population [5]. Different ventilation modes have been proposed to reduce ventilation-induced lung injuries [6, 7]. Among them, high-frequency oscillatory ventilation (HFOV) has been proven to be an efficient and safe mode of ventilation in preterm infants [8–10]. In comparison with volume targeted conventional mechanical ventilation, HFOV might reduce mortality and the incidence of bronchopulmonary dysplasia (BPD) when combined with the open lung principle [11]. The open lung principle aims to recruit the lungs and to keep them open in order to maintain optimal compliance [1, 12]. At present, lung recruitment continues to rely on oxygenation and pressure indices to assess changes in lung volume since no direct bedside tool for monitoring lung volume has been established so far [13, 14]. Stepwise oxygenation-guided lung recruitment maneuvers (LRMs) can efficiently establish and stabilize lung capacity after HFOV initiation [13–15]. Studies have further confirmed that such LRMs have a low risk of lung hyperinflation and air leak syndrome, have no adverse cerebral events (e.g., intraventricular hemorrhage) and in addition, do not compromise cardiac function up to a critical level in preterm infants [16]. Therefore, LRMs are recommended when HFOV is initiated [13, 14]. They should be repeated in situations with partial lung collapse to restore lung capacity. Although titrating mean airway pressure (MAP) every 12 h has been discussed, neither the assessment of partial lung collapse nor the time interval when to repeat LRMs are well-defined and the question when to perform LRMs during HVOF has not been addressed so far [1].

The present study explored the question on the efficiency of regularly performed LRMs in terms of cumulative oxygenation and mean airway pressure. We hypothesized that regularly performed LRMs decrease the cumulative oxygenation saturation index (OSI) during HFOV in preterm infants. The study further investigated LRMs with respect to changes in oxygenation indices and mean airway pressure.

## Methods

The trial (ClinicalTrials.gov ID: NCT04289324, 28/02/2022) was conducted at a third level neonatal intensive care unit of the Medical University of Vienna. It was approved by the local ethics committee (EK 1161/2019). Written informed consent was obtained from parents or legal guardian of each infant prior to the study participation.

This study was a single-center, open, randomized controlled trial. Preterm infants born below 28 weeks of postmenstrual age (PMA) were screened for eligibility. Infants with congenital anomalies of the heart and/or the lungs as reported in ultrasound and/or fetal MRI were excluded. Infants were enrolled in the study at the time of HFOV initiation (Fabian HFO respirator, Acutronic Medical Systems AG, Hirzel, Switzerland) and received either LRMs at regular 12-hour intervals and upon decision of the care giving team (intervention group), or LRMs only upon decision of the caregiving team (control group). HFOV is used as a primary ventilation mode for extremely preterm infants in our unit. Participants were randomly allocated with a 1:1 ratio, stratified for gender and PMA (less than 26 weeks versus equal or higher than 26 + 0 weeks) using the Randomizer for Clinical Trials tool developed at the Medical University of Graz (http://www.randomizer.at/). Patient data were collected from the medical charts: PMA at birth and at HFOV initiation, weight at birth and at HFOV initiation, day of life at HFOV initiation, the oxygenation saturation index at HFOV initiation, gender, and complete course of antenatal steroids.

### Lung recruitment maneuver

LRMs were performed in hemodynamically stable situations only and were conducted as previously described [10, 13, 14, 17, 18]: oscillatory frequency was kept at the clinically set values and the pressure amplitude was adjusted to maintain the transcutaneous pCO2 between 40 and 60 mmHg (SenTec Digital Monitor, Therwil, Switzerland). Starting at the clinically set MAP value, MAP was increased every 5 min (allowing intervals of 2–15 min upon the decision of the caregiving team) by 2 mbar (with steps of 1 mbar when greater than 20 mbar, maximum MAP was set at 25 mbar). FiO2 was reduced stepwise, keeping SpO2 within the predefined target range (88–96% or 90–96% in presence of pulmonary hypertension requiring medication). Increase of MAP was stopped when SpO2 no longer improved or FiO2 was ≤ 0.25 (MAPmax, maximum MAP during the LRM). Next, MAP was gradually decreased every 5 min (allowing intervals of 2–15 min upon the decision of the caregiving team) by 2 mbar until a sustained drop in SpO2 of ≤ 5% of the initial value at MAPmax or an absolute SpO2 value below 88% (minimum MAP 5 mbar) reaching the closing MAP. After a one-step recruitment with the known MAPmax, the final MAP was set 1–2 mbar above the closing MAP (see example in the supplemental material). LRMs were advised after the following situations: change of position (from prone to supine or vice versa), any manipulation with FiO2 increase of 0.1 or SpO2 decrease > 10% for > 5 min (e.g., suctioning, endo-tracheal tube disconnection), surfactant application, and suspected or confirmed atelectasis (e.g., diagnosed on chest X-ray). LRM was compulsory each time HFOV was initiated. LRMs (per protocol) were scheduled for 8AM and 8PM in the intervention group. If a LRM was performed 2 h prior to the schedule or was planned to be performed within 2 h after the schedule (e.g., change of position at 9AM), then the scheduled LRM was omitted. The care giving staff were instructed in how and when to perform LRMs prior to the initiation of the trial.

The primary outcome was defined as the cumulative OSI during HFOV in the study period which started with HFOV initiation and was limited to at most 7 consecutive days. The oxygenation saturation index (OSI) is determined by the product of FiO2 and MAP divided by the peripheral oxygen saturation (SpO2) and is expressed as a percentage. The cumulative OSI is determined by the sum of OSI values divided by the number of OSI values in the study period which is equivalent to the average OSI. The OSI variables were recorded every 15 min in the electronic patient data management system (ICCA, Philips Healthcare, Amsterdam, Netherlands) and structurally obtained. Data was assessed by a clinical expert for plausibility. Outliers as well as LRMs were not considered for analysis.

Secondary outcome variables were the number of LRMs, the duration of mechanical ventilation, oxygenation parameters (SpO2, FiO2, S/F-ratio) and MAP averaged over HFOV time, mortality and morbidity including BPD as defined by the requirement of respiratory support and supplemental oxygen at PMA of 36 weeks, severe intraventricular hemorrhage (IVH > grade II, as seen on cranial ultrasound scans), pneumothorax (PTX, as seen in the chest X-ray), and pulmonary interstitial emphysema (PIE, as described in the radiologic results).

All LRMs performed in the study period were analyzed with respect to the change of OSI prior and after LRM in dependence of the oxygen requirement and MAP at the start of the LRM, of the duration of the LRM, and of the time difference between two adjacent LRMs.

### Sample size calculation

Zannin et al. observed that LRMs after HFOV initiation reduced the OSI of approximately 25%, from 6.15 to 4.55 (SD approximately 1.5) on average [13]. We assumed that this reduction extends to the cumulative OSI during HFOV when performing LRM at regular intervals (null-hypothesis: average OSI between intervention and control group are not different). Based thereupon, 15 infants need to be enrolled in each group to attain 80% power for detecting a difference of 25% in cumulative OSI between the groups (at a two-sided alpha level of 5%). The sample size is based on calculations using G*Power 3.1.9.2 (University of Kiel, Germany) with the following inputs (t-tests): difference between two independent means (two groups); tails = 2; effect size d = 1.067; alpha = 0.05, power (1-β error probability) = 0.8, allocation ratio N2/N1 = 1.

### Statistical analysis

For hypothesis testing of the primary outcome (cumulative OSI), the unpaired t-test was used. For evaluating differences of continuous parametric and continuous non-parametric variables between the intervention and the control group the unpaired t-test and the Mann-Whitney U-test were used, respectively. The difference of the dichotomy variables between the groups was tested using the Fisher’s exact test. MAP, S/F ratio, and OSI before and after LRM for all LRMs were compared using the paired t-test. Differences were considered statistically significant for *p* < 0.05. Multivariable logistic regression analysis to determine independent risk factors for BPD was performed using cumulative OSI over HFOV time, number of LRMs, PMA at birth, birth weight, ventilation days, and antenatal steroids as risk factors for death or moderate to severe BPD (Grade II-III(A)) [19]. Linear regressions between change in OSI and FiO2 levels were performed to assess differences between LRM indications. Analyses of the secondary outcome variables were considered as hypothesis generating and therefore no adjustment for multiple testing was performed. Statistical analysis was performed with R version 4.0.2 (The R Foundation for Statistical Computing, Vienna, Austria) using RStudio version 1.3.1056 (RStudio Team 2020, RStudio, Inc., Boston, MA).

## Results

### Study participants

**Fig. 1:**
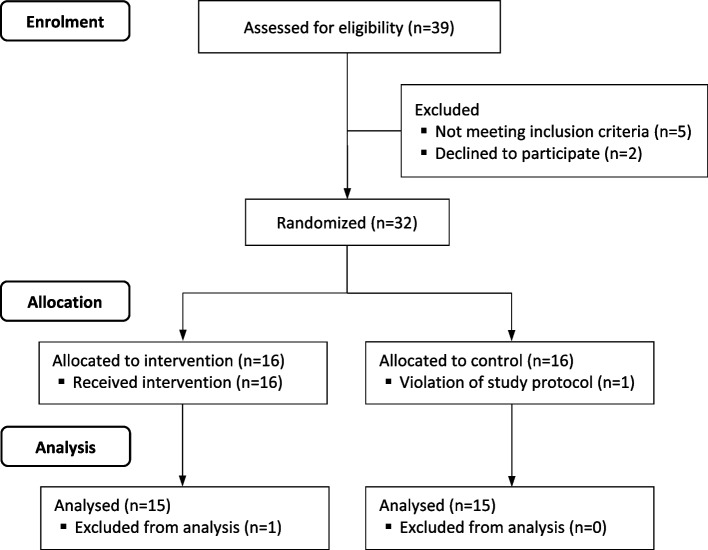
CONSORT Flow diagram

The study was conducted from March 2020 to June 2021. Thirty-nine extremely preterm infants were screened for eligibility prior to HFOV initiation. In 2 cases, parents refused trial participation. Five patients did not receive HFOV. Thirty-two preterm infants were randomized at HFOV initiation, 16 allocated in each group. Two participants were excluded during the trial, one in each group. The infant excluded from the control group was on HFOV less than 12 h and no LRM did take place violating the trial protocol. The infant in the intervention group was excluded due to technical problems with data monitoring. After exclusion, 15 participants were analyzed in each group as presented in the flow diagram (Fig. 1).

The baseline characteristics of the study participants are presented in Table 1. The PMA at the time of study initiation was slightly different between the intervention and the control group (*p* = 0.03). In each group, only 1 participant was born at a PMA greater than 26 weeks. All participants received surfactant in the first hour of life.

**Table 1:** Baseline characteristics

Characteristics		Intervention (*n* = 15)	Control (*n* = 15)	*P*-value*
GA at birth, mean (SD), [weeks + days]		23 + 6 (0 + 5)	24 + 4 (0 + 6)	0.06
Weight at birth, mean (SD), [g]		650 (115)	615 (95)	0.38
Antenatal steroids (complete administration), n (%)		9 (60)	8 (53)	0.79
Male, n (%)		9 (60)	10 (67)	0.45
Day of life at HFOV start, mean (SD), [d]		3 (4)	5 (6)	0.23
GA at HFOV start, mean (SD), [weeks + days]		24 + 2 (0 + 6)	25 + 1 (1 + 1)	0.03
Weight at HFOV start, mean (SD), [g]		650 (115)	615 (105)	0.36
OSI at HFOV start, mean (SD)		4.09 (1.94)	5.87 (3.64)	0.11
Main cause of respiratory failure				
Severe respiratory distress syndrome, n (%)		11 (73.3)	7 (46.7)	0.26
Sepsis, n (%)		1 (6.7)	4 (26.7)	0.33
Pneumonia, n (%)		0 (0)	2 (13.3)	0.48
Necrotizing enterocolitis, n (%)		2 (13.3)	1 (6.7)	1.0
Severe pulmonary hypertension, n (%)		1 (6.7)	1 (6.7)	1.0

*HFOV* high frequency oscillation ventilation, *OSI* oxygenation saturation index, *PMA* postmenstrual age

*Mann-Whitney U test for nonparametric data and Fisher’s exact test for categorical data

### Primary outcome

The cumulative OSI value during HFOV was lower in the intervention group, however, no statistical significance could be demonstrated (4.95 versus 5.30, *p* = 0.61). The percentage of OSI values that were excluded (outliers or recordings during LRMs) from the calculation of the cumulative OSI amounted to 1.9 (SD 2.4) and were similar between both groups, 2.0 (SD 2.1) in the intervention group and 1.8 (SD 2.7) in the control group (*p* = 0.83). Considering the excluded OSI values for analysis did not change the primary outcome: 4.99 (SD 1.75) in the intervention group and 5.40 (SD 2.24) in the control group (*p* = 0.58). We point out that the average number of performed LRMs within 12 h in the intervention group was only slightly greater than in the control group (1.33 versus 1.11, *p* = 0.13), see Table 2.

### Secondary outcome

None of the secondary outcome parameters showed any statistically significant difference between groups, see Table 2. Logistic regression analysis showed cumulative OSI over HFOV time (*p *= 0.016) to be a significant independent risk factor for death or moderate to severe BPD while number of LRMs, PMA, birth weight, ventilation days, and antenatal steroids were not. The mean (SD) of cumulative OSI between respiratory failure due to severe RDS (*n* = 18) and non-RDS (*n* = 12) amounted 4.9 (2.1) and 5.4 (1.5), respectively (*p* = 0.46).

### Lung recruitment maneuver

The number of LRMs performed during the study period amounted to 338. We observed at most a slight and transient decrease in blood pressure during the inflation limb of LRMs. FiO2 clearly improved, whereas the change in MAP was less pronounced. The mean (SD) FiO2 improvement for all LRMs amounted to 11% (16%). No significant difference in FiO2 improvement was found among groups of different LRM indication. The largest improvement in FiO2 occurred when LRM took place after surfactant application and after changing the patient’s position with an average FiO2 reduction of 14% and 13%, respectively, see Table 3.

**Fig. 2:**
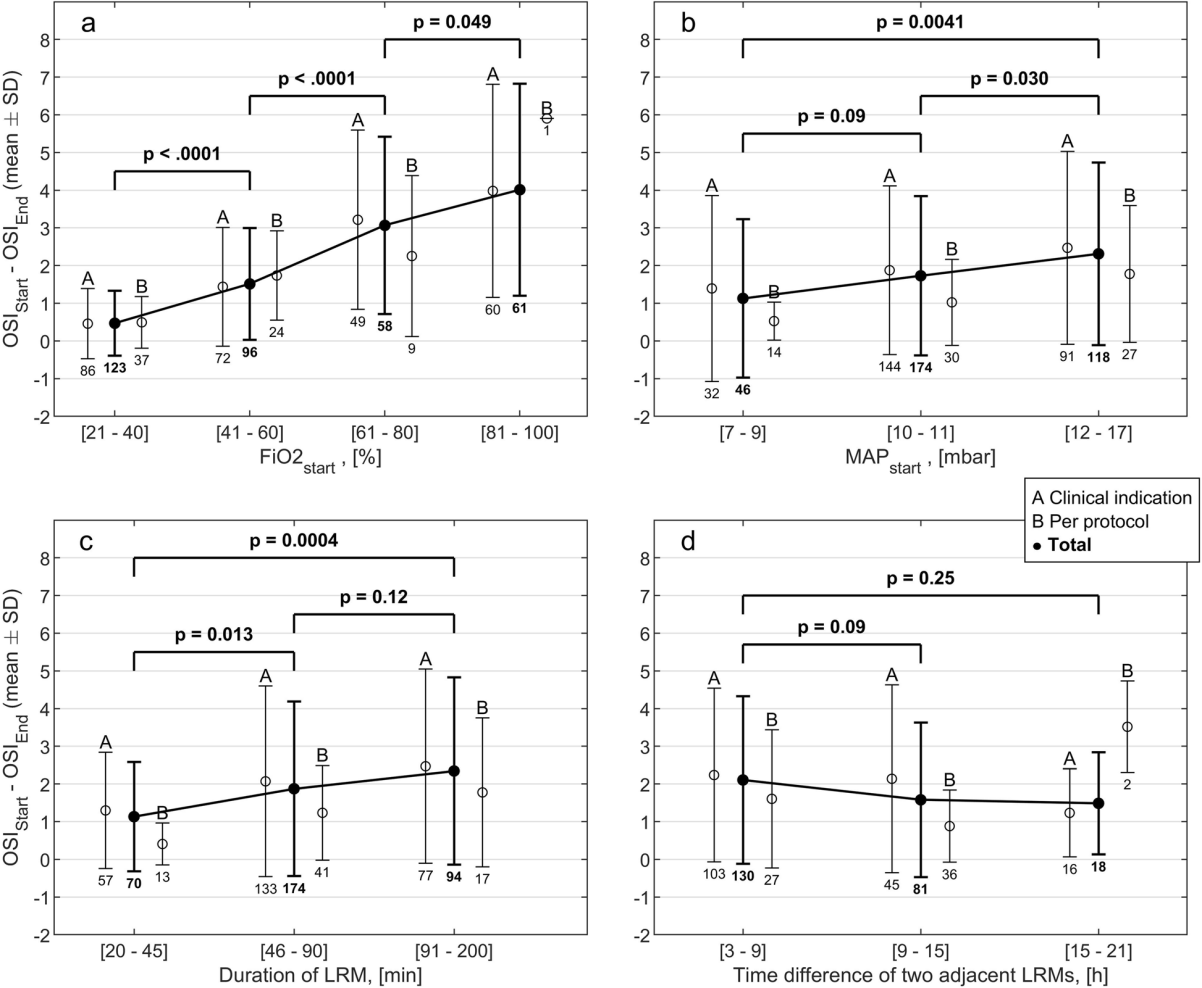
Change of oxygenation saturation index (mean difference between start and end of LRM) in dependence of (**a**) different FiO_2_ levels at the start of LRM, (**b**) MAP levels at the start of LRM, (**c**) the duration of LRM, and (**d**) of the time difference between two adjacent LRM for all (bold), clinically indicated (A), and clinically not indicated (B, intervention only) LRMs. The number of LRMs is displayed under each bar. The unpaired t-test was applied to calculate *p*-values for all recruitment maneuvers in the intervals specified below each figure. OSI, oxygenation saturation index; FiO_2_, fraction of inspired oxygen; LRM, lung recruitment maneuver; MAP, mean airway pressure

When considering FiO2 and MAP prior to LRM, we observed that FiO2 values above 0.6 exhibit the highest OSI reduction of 3.6 (SD 2.6) on average (Fig. 2, a). For MAP levels below 10 mbar, the OSI reduction amounted only to 0.5 (SD 0.5) on average (Fig. 2, b).

**Table 2:** Results

Results		Total (*n* = 30)	Intervention (*n* = 15)	Control (*n* = 15)	P*
**Primary outcome**					
Cumulative OSI, mean (SD)		5.13 (1.90)	4.95 (1.72)	5.30 (2.08)	0.61
**Secondary outcome**					
Average number of recruitments in 12 h, mean (SD)		1.22 (0.40)	1.33 (0.22)	1.11 (0.51)	0.13
Average number of recruitments, median (min, max), n		11 (1,21)	13 (3, 21)	8 (1, 21)	0.25
Duration of CMV in study period, median (min, max), h		7.0 (0, 108)	7.3 (0, 55)	6.8 (0, 108)	0.87
Duration of HFOV in study period, median (min, max), h		121 (30, 173)	127 (30, 173)	104 (34, 161)	0.56
Total duration of CMV, median (min, max), h		82 (0, 850)	160 (0, 551)	73 (0, 850)	0.27
Total duration of HFOV, median (min, max), h		129 (31, 445)	131 (31, 445)	107 (34, 254)	0.43
Average^a^ S/F ratio, mean (SD)		256 (68)	260 (68)	251 (69)	0.71
Average^a^ SpO_2_, mean (SD) [%]		91.4 (0.8)	91.3 (0.9)	91.5 (0.7)	0.51
Average^a^ FiO_2_, mean (SD) [%]		0.43 (0.14)	0.42 (0.13)	0.43 (0.16)	0.78
Average^a^ MAP, mean (SD) [mbar]		10.8 (1.2)	10.5 (1.4)	11.1 (1.0)	0.18
Maximum MAP in LRM, median (min, max), [mbar]		19 (11, 25)	19 (11, 25)	18 (13, 24)	0.35
Survival, n (%)		21 (70)	10 (67)	11 (73)	1.0
BPD (I-III)^b^, n (%)		18 (60)	10 (67)	8 (53)	0.71
BPD (I-III)^b^ in survivors, n (%)		21 (76)	9 (90)	7 (64)	0.31
BPD (II-III)^b^ in survivors, n (%)		5 (24)	4 (40)	1 (9)	0.14
Pneumothorax, n (%)		5 (17)	3 (20)	2 (13)	1.0
Severe intraventricular hemorrhage, n (%)		7 (23)	5 (33)	2 (13)	0.39
Pulmonary interstitial emphysema, n (%)		4 (13)	2 (13)	2 (13)	1.0

*BPD* Bronchopulmonary dysplasia, *CMV* conventional mechanical ventilation, *h* hours, *HFOV* high frequency oscillation ventilation, *LRM* lung recruitment maneuver, *MAP* mean airway pressure

^a^mean over HFOV duration in study period

^b^BPD as defined in [19]. RM, recruitment maneuver during HFOV duration in study period

*The unpaired t-test for continuous variables, the Fisher’s exact test for categorical data, and the Mann-Whitney U test for nonparametric data

**Fig. 3:**
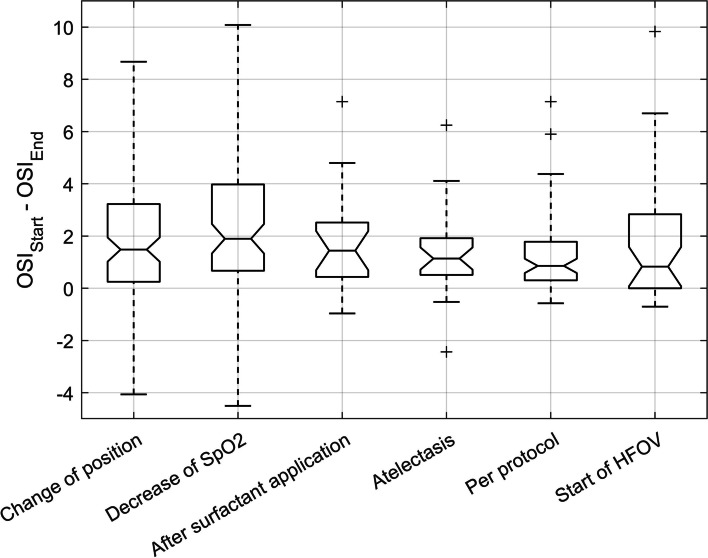
Boxplot displaying changes of the oxygenation saturation index after lung recruitment for different groups of lung recruitment indication. On each box, the central line indicates the median, and the bottom and top edges represent the 25th and 75th percentiles, respectively. The whiskers extend to the most extreme data points within two times the interquartile range. The ‘+’ symbol indicates the outliers. No statistically significant difference could be found among the groups

LRMs with a duration of less than 45 min resulted in a mean OSI reduction of 1.1 (SD 1.4) whereas a duration of more than 90 min resulted in significantly higher OSI reduction of 2.3 (SD 2.5, *p* < 0.001), (Fig. 2, c). ). In comparison with LRMs with a duration of more than 90 min, those with a duration of less than 45 min had a significantly lower number of recruitment steps (median of 8 versus 15, *p* < 0.001) and a significantly shorter duration of recruitment steps (mean of 4.0 versus 7.6 min, *p* < 0.001). LRMs performed after 3 to 9 h of the previous LRM showed a mean OSI reduction of 2.1 (SD 2.2), whereas LRMs after 9 to 15 h of the previous LRM showed a mean OSI reduction of 1.6 (SD 2.1). However, this finding did not reach statistical significance (*p* = 0.09), (Fig. 2, d). No remarkable difference in OSI reduction between LRM indications could be observed (Fig. 3).

**Table 3:** Lung recruitment maneuvers grouped according indications

Indication	N (%)	D [min]^b^	MAPmax	MAP start	MAP end	FiO2 start	FiO2 end
Start HFOV	35 (9)	75 (40)	18 (3)	11.2 (1.8)	10.5 (2.2)	50 (26)	38 (18)*
Surfactant application	19 (5)	68 (40)	19 (3)	11.2 (1.6)	11.4 (1.4)	55 (22)	41 (16)*
Change of position	102 (26)	80 (47)	20 (3)	11.0 (1.4)	10.9 (1.5)	61 (26)	48 (23)*
Decrease of SpO_2_^a^	84 (22)	63 (44)	19 (4)	11.0 (1.8)	10.5 (1.9)	56 (25)	48 (22)*
Atelectasis	27 (7)	57 (42)	18 (2)	10.8 (1.2)	10.7 (1.4)	57 (24)	47 (18)*
Per protocol	71 (18)	72 (40)	18 (3)	10.9 (1.8)	10.4 (1.8)	44 (17)	36 (14)*
All	388	70 (43)	19 (3)	11.0 (1.6)	10.8 (1.6)	56 (24)	45 (20)*

*HFOV* high frequency oscillatory ventilation, *D* Duration, *FiO2* fraction of inspired oxygen in [%], *MAP* mean airway pressure in [mbar], *MAP max* maximum MAP of the lung recruitment maneuver

^a^Manipulation (e.g., suctioning, disconnection of the endo-tracheal tube) with FiO2 increase of > 0.1 or SpO_2_ decrease > 10% for > 5 min

Numbers represent mean (SD) else indicated. ^b^ median (IQR), * *p* < 0.001 (paired t-test)

## Discussion

### Primary outcome

This randomized controlled trial is the first study that analyzed repeatedly performed LRMs during HFOV in extremely preterm infants. The primary cause for respiratory failure in this cohort was severe RDS. The study was targeting 12-hourly scheduled versus clinically indicated lung recruitment maneuvers. The findings did not result in a significant change in the number of lung recruitment maneuvers, and as a consequence, no difference in oxygenation was observed between the study groups. Our observation suggests that the course of HFOV lead to situations of reduced lung volume that require LRMs at least once every 12 h. As a result, the policy of performing LRM based solely on the clinical judgement seems not to be inferior as compared to a fixed 12-hour recruitment regime. We conclude that imposing scheduled lung recruitment maneuvers twice a day during HFOV is not needed in a lung protective setting using lung recruitment maneuvers for preventing prolonged lung collapse in preterm infants. The study was part of a quality improvement process in which we aimed to standardize LRMs during HFOV. Physicians received appropriate training regarding LRMs prior to the study initiation. Before standardization, LRMs were performed occasionally and only in situations of severe respiratory deterioration and not in the sense of a lung protective strategy. Standardized indication of LRMs could have led to an increased awareness for lung protective strategies among the caregiving team. Both, the awareness for lung protective strategies and the implementation of guidelines for LRMs might account for the lack of difference in both study groups.

### Lung recruitment maneuver

LRMs aim to (re)-open collapsed lung areas that are poorly aerated avoiding overdistention with the ultimate goal to prevent ventilation induced lung injury [20, 21]. The important question in this context refers to the recruitability of lung units. Assessing recruitability of the lung is difficult in clinical practice. We have observed that performing LRMs at high FiO2 levels was most beneficial in terms of OSI reduction which is indicative of an improvement in lung volume. Hence, FiO2 might serve as an predictor for the recruitability of the lung. However, we acknowledge that oxygenation varies with systemic hemodynamics in respiratory failure, and therefore does not always reflect alveolar recruitment [22]. Reduction in OSI for higher MAP levels prior to the LRM was less pronounced. High MAP levels bear the risk of overdistention and regularly performed LRMs might overcome this problem by eventually de-recruiting overdistended lung areas. LRMs with a duration longer than 90 min showed a significantly larger OSI reduction than short LRMs. This observation is in accordance with the findings by Thome et al., namely that alveolar recruitment may take up to 25 min after MAP changes [23]. No clear benefit in OSI reduction has been found when repeating LRMs in less than 9 h versus more than 15 h (Fig. 2, d). It seems that clinical indication for performing LRMs is more effective than LRMs per protocol (Fig. 2). This might be explained by the fact that FiO2 at LRM initiation was lower for LRMs per protocol than for clinical indication (Table 3). However, a difference in OSI reduction between LRM indications could not be detected (Fig. 3). We further observed, that LRMs were most often performed after positional change (Table 3). Changing the patient’s position, in particular from supine to prone, is a common procedure in ventilated infants and a key element in lung protective strategies in patients with acute respiratory distress syndrome [24–26]. Recruiting the lung after positional change seems to be more beneficial in terms of OSI reduction than performing LRMs for other reasons (Table 3). This might be explained by the fact that LRMs are thought to potentiate the recruiting effect induced by changing the patient’s position [27]. Overall, we observed that OSI was markedly reduced by LRM when FiO2 was high regardless of the indication to perform the LRM. However, we need to mention that it remains unclear to what extent an episodic reduction in OSI has a lung protective effect. Our observations of the effect on OSI by LRMs remain hypothetical and further research is needed for confirmation.

This study has some limitations. Firstly, changes in lung volume during the recruitment maneuvers were not measured directly and the cumulative OSI might not be an adequate parameter to assess lung volume. However, to date, no bedside parameter other than oxygenation is established to guide LRMs. Moreover, data suggest that a reduction in cumulative FiO2 may reduce the incidence of BPD [28]. In addition, the average product of FiO2 and MAP over ventilation time has been identified as an independent predictor of BPD and necrotizing enterocolitis [29]. OSI serves as an alternative to the product of FiO2 and MAP, taking into account different SpO2 targets [30]. Secondly, the assumption that the 25% reduction in OSI after a single LRM as reported by Zanin et al. would expand to the cumulative OSI during HFOV was speculative [13]. We found an average reduction of OSI for all LRMs of 22% confirming the results by Zanin et al. but could not find any difference in the cumulative OSI between the groups since the average number of LRMs did not differ. However, the cumulative OSI appeared to be an independent predictor for BPD which supports its use for sample size estimation. Thirdly, we chose to perform LRMs by clinical indicators for lung collapse only. Therefore, we were not able to report the main cause of lung collapse or increasing FiO2 prior to every LRM which could have been helpful to classify the respiratory condition.

## Conclusion

During HFOV, stepwise oxygenation-guided LRMs were performed at least once in 12 h when using clinical indicators for collapsed lung units in extremely preterm infants. Imposing scheduled LRMs twice a day did not improve oxygenation. LRMs seem to be most effective at high oxygen requirement and should be considered particularly after changing the patient’s position facilitating the increase of lung compliance.

## Supplementary Information

**Additional file 1.**

## Data Availability

The data will be made available from the corresponding author on reasonable request.

## Abbreviations

BPD: Bronchopulmonary dysplasia
FiO2: Fraction of inspired oxygen
HFOV: High-frequency oscillatory ventilation
IVH: Intraventricular hemorrhage
LRM: Lung recruitment maneuver
MAP: Mean airway pressure
OSI: Oxygen saturation index
PMA: Postmenstrual age
PTX: Pneumothorax
PIE: Pulmonary interstitial emphysema
S/F: Oxygen saturation to fraction of inspired oxygen ratio
SpO2: Peripheral oxygen saturation
