# Supplementary material for: Regular lung recruitment maneuvers during high-frequency oscillatory ventilation in extremely preterm infants: a randomized controlled trial
Source: BMC Pediatr. 2022 Dec 12;22:710. doi: 10.1186/s12887-022-03780-7 (PMC9743585; doi:10.1186/s12887-022-03780-7)

Supplementary figure: Example of a stepwise oxygenation guided lung recruitment maneuver during high frequency oscillatory ventilation. MAP, mean airway pressure [cmH2O]; Vt, tidal volume [mL]; Amp, amplitude [cmH2O].

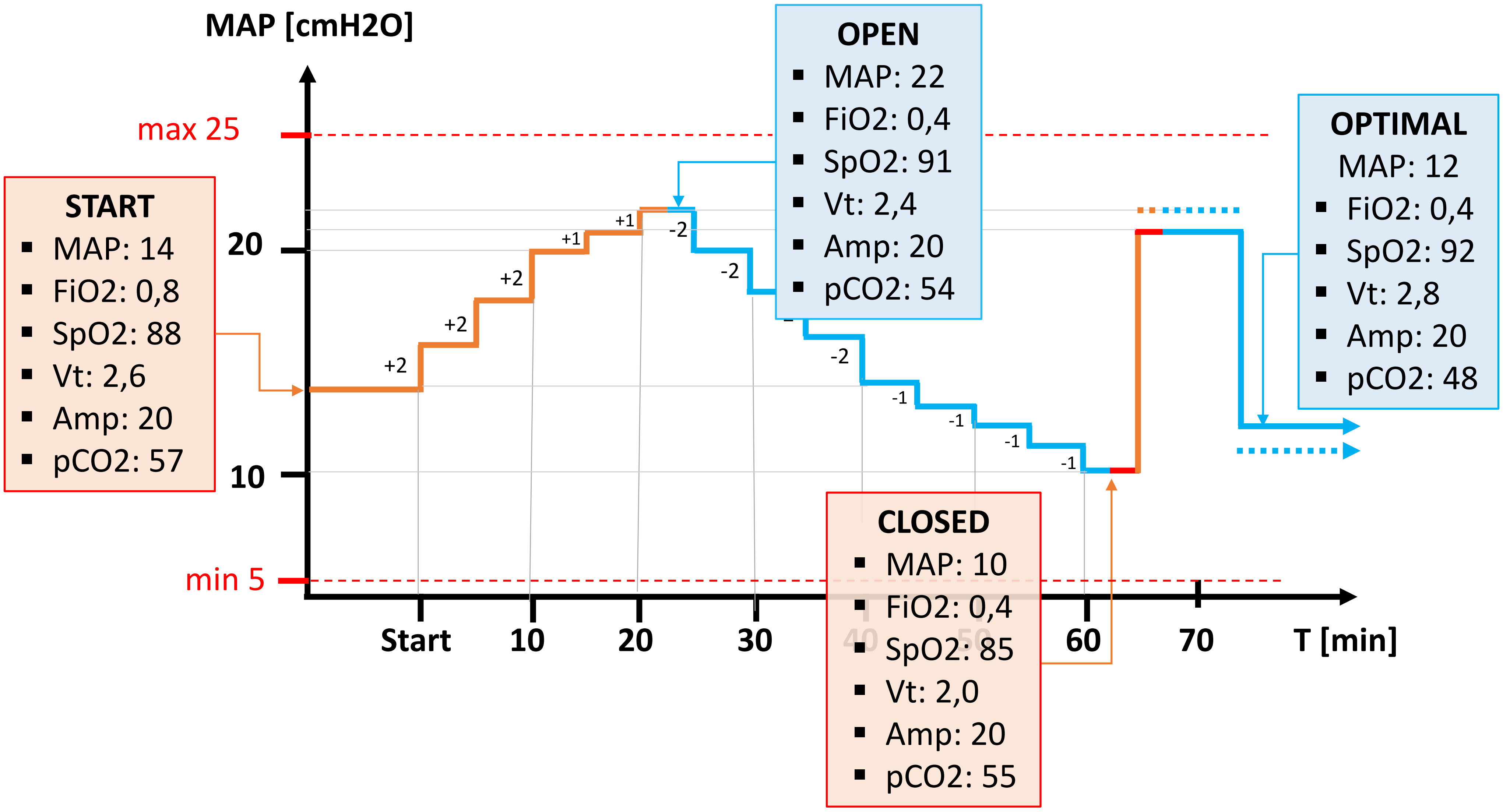

Supplement: Supplementary file 1 — Additional file 1. [file 12887_2022_3780_MOESM1_ESM.pdf]
